# Explaining Disparities in Use of Skilled Birth Attendants in Developing Countries: A Conceptual Framework

**DOI:** 10.1371/journal.pone.0154110

**Published:** 2016-04-22

**Authors:** Patience A. Afulani, Cheryl Moyer

**Affiliations:** 1 Department of Obstetrics, Gynecology, & Reproductive Sciences, School of medicine, University of California San Francisco, San Francisco, California, United States of America; 2 Departments of Learning Health Sciences and Obstetrics & Gynecology and Global REACH, University of Michigan, Ann Arbor, Michigan, United States of America; National Institute of Health, ITALY

## Abstract

Despite World Health Organization recommendations that all women deliver with a skilled birth attendant (SBA), research continues to demonstrate large disparities in use of SBAs by socioeconomic status (SES). Yet few quantitative studies empirically examine the factors underlying these disparities, due in part to the fact that current models do not provide clear pathways—with measurable mediators—for how distal factors like SES may affect maternal health-seeking behaviors like delivering with SBAs. We propose the Disparities in Skilled Birth Attendance (DiSBA) framework for examining the determinants of use of SBAs. We posit that three proximal factors directly affect use of SBAs: perceived need, perceived accessibility of maternal health services, and perceived quality of care. Distal factors like SES affect use of SBAs indirectly through these proximal factors, and the effects can be measured. We test the assumptions of the DiSBA framework using data from the Ghana Maternal Health Survey. The analytic techniques we use include logistic regression with mediation analysis to examine the intervening effects. We find that our proxies for perceived access, perceived need, and perceived quality of care account for approximately 23% of the difference between women with no education and those with primary school education, and about 55% of the difference between women in the lowest wealth quintile and those in the middle wealth quintiles. This study suggests that proximal factors are worthy of increased attention in terms of measurement, data collection, analysis, programmatic efforts, and policy interventions, as these factors are potentially more amenable to change than the distal factors. The effects of proximal factors are also likely context specific, thus sufficient understanding in different contexts is essential to developing appropriate interventions.

## Introduction

Skilled attendance at delivery is a critical intervention to reduce maternal mortality [1,2], given that approximately three quarters of maternal deaths occur from complications during labor, delivery, and the first 24 hours postpartum [3]. These complications are difficult to predict, but can be effectively managed and deaths averted if they are recognized and treated promptly. Thus, the World Health Organization recommends every delivery ought to be overseen by a *skilled birth attendant* (SBA)—a health professional who can identify and manage normal labor and delivery; and identify and treat complications or provide basic care and referral [1,3,4]. Unfortunately, the proportion of deliveries by SBAs is still below recommended levels. In Sub-Saharan Africa (SSA), about half of births are assisted by SBAs—with wide disparities by socioeconomic status (SES) [5–7]. Even in countries where antenatal care (ANC) is common, a large proportion of deliveries occur at home without the help of a SBA [8,9].

Ghana exemplifies the experience of many countries in SSA. The maternal mortality ratio in Ghana is 380 maternal deaths per 100,000 live births [10]. More than 95% of Ghanaian women have at least one ANC visit during pregnancy, and about 80% attend the recommended four or more visits [11–14]. In 2008, only about half of births were assisted by a SBA, with wide disparities by SES. Only 36% of births among women with no education were assisted by SBAs, compared to 92% among those with secondary education or more; and 24% among women in the poorest wealth quintile compared to 95% among those in the richest quintile [12]. The 2011 UNICEF multiple indicator cluster survey and the 2014 Ghana Demographic and Health Survey (GDHS) key findings show the proportion of births assisted by SBAs increased to 63% in 2011 and to 75% in 2014, but the SES disparities still remain [13,14]. These statistics raise two questions that motivate this research: (1) What accounts for the disparity in ANC attendance and use of SBAs in Ghana? (2) What accounts for the SES disparities in use of SBAs within the country?

Many studies have examined the determinants of use of SBAs or deliveries in health facilities, with a number of reviews on the topic [15,16,5,6]. These reviews all show large socioeconomic and rural/urban disparities in skilled attendance, with higher education, higher wealth, and urban residence consistently associated with higher use of SBAs. In Africa, even a primary education is associated with higher utilization compared to no education. Women in the second lowest wealth quintile have higher utilization than those in the lowest wealth quintile. These disparities persist even after controlling for other factors, many of which have been examined in qualitative studies [17–20]. Few quantitative studies have, however, empirically examined the factors underlying these disparities. This is likely because current models do not provide clear pathways—with measurable mediators—for how distal factors like SES may affect maternal health-seeking behavior. The goal of this paper is to help bridge this gap.

In this paper, we propose a new framework—the Disparities in Skilled Birth Attendance (DiSBA) framework—that explicitly lays out potential mediating pathways through which distal factors like SES may affect use of SBAs. We then empirically examine factors underlying SES disparities in SBA use in Ghana, as well as the gap between ANC attendance and SBA use. In addition, we identify gaps in the existing data that limit our understanding of the sources of disparities in service utilization and make recommendations for future research.

### Existing frameworks to understand use of skilled attendants

The DiSBA framework draws on prior research on maternal mortality and the determinants of use of maternal health services. In particular we draw on three prior models [21,15,5]: McCarthy and Maine’s (1992) framework for analyzing the determinants of maternal mortality; the three delays model by Thaddeus and Maine (1994), which posits three delays—the delays to seek, reach, and receive care—that lead to maternal mortality from the onset of an obstetric complication; and the recent expansion of the three delays model by Gabrysch and Campbell (2009) to include preventive obstetric care.

McCarthy and Maine’s comprehensive framework highlights the influence of contextual factors yet it does not illustrate the complex interactions amongst the intermediate determinants. Thaddeus and Maine’s three delays model focuses on pregnant women and their care-seeking behavior when they experience a complication. This model has been widely used, yet it implies a single pathway for the effect of SES—a single arrow from socioeconomic/cultural factors to the delay to seek care. But socioeconomic/cultural factors can also affect the delay to reach and receive care through a number of pathways. Gabrysch and Campbell’s model includes care seeking for uncomplicated pregnancies and partially addresses the multiple pathways for socioeconomic/cultural factors by separating out economic factors from sociocultural factors, with sociocultural factors affecting to the delay to seek care, and economic and physical accessibility affecting the delays to seek, identify, and reach care. However, certain sociocultural factors may still lead to other delays besides delays in deciding to seek care. For example, sociocultural factors related women’s autonomy can potentially affect the time to reach care, given situations where women cannot travel freely or are not allowed to use certain types of transportation, which may be the most readily available form of transportation [15,22].

None of these models address the effect of socioeconomic factors on the delay in receiving adequate and appropriate treatment. SES can impact the timeliness and quality of care received in several ways. First, in settings where patients are expected to buy supplies and medication upon reaching the health facility, higher SES women who can afford such supplies are much more likely to receive prompt attention. It has also been demonstrated that higher SES women are treated differently than lower SES women, suggesting that quality of care may be better for women with means [23–25]. In addition, higher SES women have the means to choose facilities—such as private, non-government facilities—that may offer better quality of care [26–29]. These factors will in turn affect their perceptions of the quality of care, which will affect future decisions of whether or not to return for care [5,24,30].

These examples suggest a complex interaction between socioeconomic factors and the phases of delays that are not explicitly illustrated in the three delays models. Our framework builds on the strengths of these earlier models and attempts to address their limitations to provide an integrated, yet simple, conceptual framework for understanding the sources of disparities in the use of SBAs. In addition, we draw on the health belief model, which posits that people are more likely to act if they perceive a need (based on their perceived susceptibility, perceived etiology, and severity of the condition), perceive that benefits of the action outweigh the barriers; and have cues to action and reinforcements [31].

### The Disparities in Skilled Birth Attendance (DiSBA) framework

The main premise of the DiSBA framework is that the decision to use maternal health (MH) services, including use of SBAs, is based on three factors: *perceived need* for care, *perceived accessibility* (physical and financial) of the service; and *perceived quality of the care*. We refer to these three factors as the *proximal determinants* of use of maternal health services. SES and place of residence, the most common determinants of service use, are *distal factors* that affect use through these three proximal factors.

Perceived need is influenced by women’s current health status (e.g., having a pregnancy complication and the type and severity of the complication), reproductive factors (including age and parity), prior health status or pregnancy complications, health knowledge (general and specific to pregnancy), as well as by unknown factors that influence the development of pregnancy complications [21]. In addition, perceived need is shaped by socioeconomic and sociocultural factors.

Perceived accessibility is influenced by actual accessibility (physical access and cost of services) [15], as well as by socioeconomic factors, and may be modified by illness factors. As noted in the three delays model, accessibility has a dual role: it indirectly influences the decision to use the service through perceived accessibility and directly influence reaching the service once the decision to use the service has been made [15]. Perceived quality is based on an assessment of quality of care from a personal previous experience, or the experience of others [15,32]. The assessment of quality can be based on the structure, process (technical or interpersonal) or outcome of care as described by Donabedian (1988), though it may be dominated by the interpersonal aspects of care; and can be based on any type of prior encounter with the health system [15,33,34]. In addition, perceived quality of care is influenced by socioeconomic and sociocultural factors [30]. All the factors are influenced by larger contextual factors in which the health system and sociocultural factors are embedded.

This paper provides a framework to guide questionnaire development, data collection, and quantitative analysis of factors that mediate SES disparities in use of SBAs. The simplicity of the model is not to undermine the numerous factors associated with use of SBAs in the literature, but to provide a way of thinking about the key predictors. It will help maternal health researchers to move beyond simply including covariates in regression models to thinking about what role the covariates are playing.

**Fig 1:**
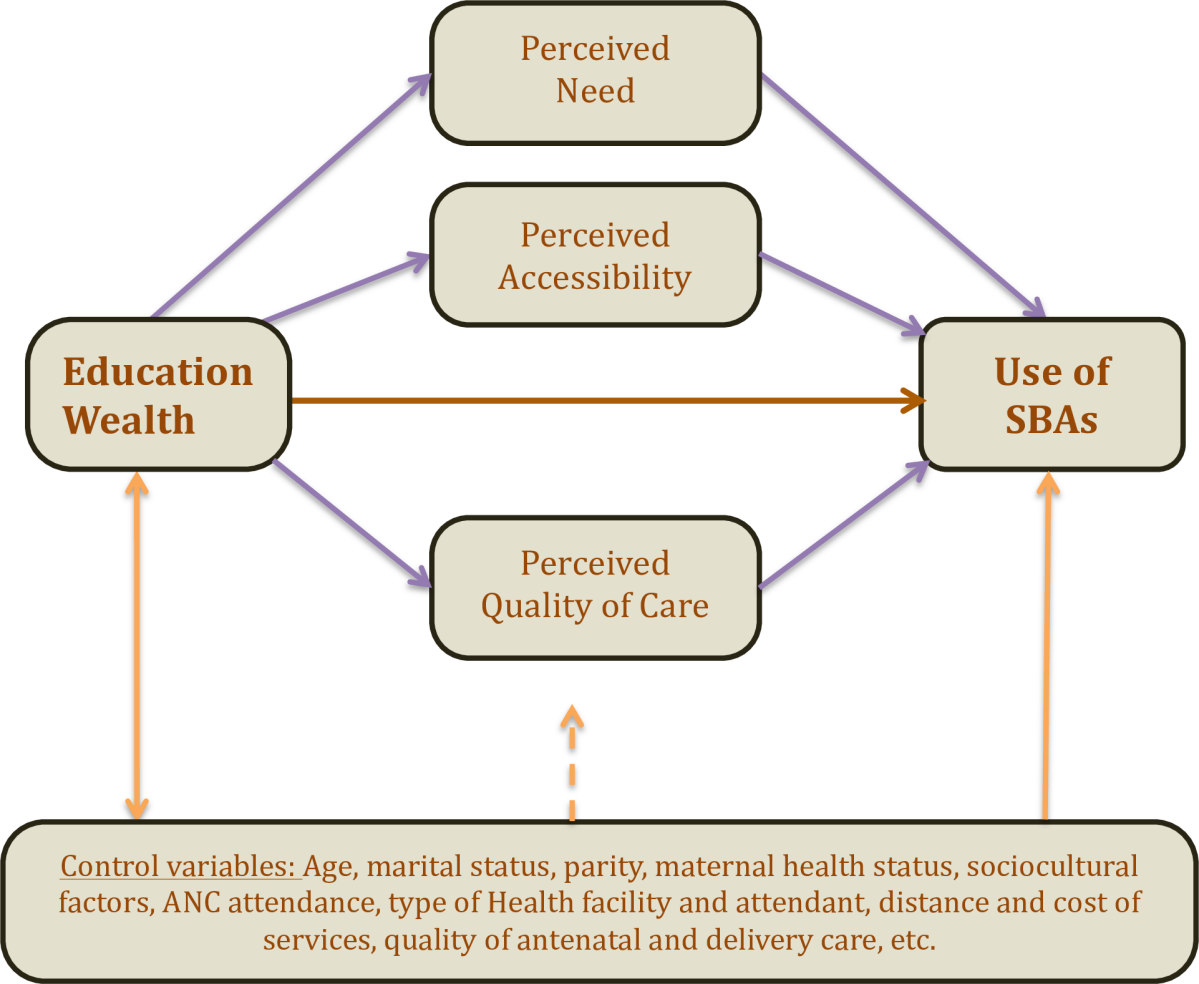
The Disparities in Skilled Birth Attendance (DiSBA) Framework, showing mediated pathways for socioeconomic status.

The DiSBA framework is illustrated in Fig 1. The key assumption of the DiSBA framework is that SES differences in perception of need, access, and quality of care account for the SES disparities in use of SBAs.

Depending on the purpose of the research, the context, and type of data available, we may pay greater attention to one or more proximal factors. It is our hope, however, that the framework will guide work from the conceptualization phase of projects that involve primary data collection and ensure that we collect data on all the proximal factors, in addition to data on the factors that influence them, to ensure more complete analysis. For the case of Ghana, where ANC attendance is much higher than use of SBAs, with large SES disparities in use of SBAs, but not in ANC attendance; and where quality of care has been mentioned as a reason for non-use of SBAs [35–37] we ask three questions:

1. Does quality of ANC predict use of SBAs among women who attend ANC at least once during pregnancy?
2. Does quality of ANC explain some of the SES disparities in the use of SBAs?
3. Compared to the other proximal determinants, what is the relative contribution of ANC quality to the SES disparities in the use of SBAs?

We focus on ANC quality because the only measures of quality in our data are from ANC. Also, since the effect of quality of care on the decision to use a SBA must be from a prior encounter (personal or vicarious) with the health system, looking at the effect of ANC quality during pregnancy on the delivery provider for that pregnancy captures this temporal ordering.

## Methods

### Data

The data for this analysis are from the 2007 Ghana Maternal Health Survey (GMHS). The survey was conducted by the Ghana Statistical Service and the Ghana Health Service with technical assistance from Macro International, and has been described in detail elsewhere [38]. Based on a multistage cluster design, households were randomly selected from all regions of Ghana and household and women’s questionnaires were administered face-to-face by trained interviewers. Verbal consent was obtained from respondents. The response rate was 99% at the household level and 98% for the individual women, with 10,858 completed household interviews and 10,370 individual interviews with women aged 15–49 years [38].

Unlike the Ghana Demographic and Health Survey (GDHS) [8], the GMHS collected health service utilization data for all women who had a birth (live or still birth, not only live births) in the five years preceding the survey (N = 5,088, or 49% of all women interviewed); this is the base sample for the analysis. The analytic sample consists of 5,042 women (99.1% of the base sample) because 46 observations are missing on key study variables. The main analysis is further restricted to women who had at least one ANC visit during their last pregnancy, since quality of ANC cannot be measured for women who did not have any ANC. Ninety seven percent (N = 4,868) of women in analytic sample had at least one antenatal visit. The full analytic sample is examined in sensitivity analysis.

This study was granted an exemption under the University of California, Los Angeles Institutional Review Board exemption category 4 for research involving the study of existing data. Ethical approval for the GMHS was however obtained from the Ghana Health Service Ethical Review Committee.

### Variables

#### Dependent variable: Delivered with a skilled birth attendant or not

Women in the survey were asked: “When you gave birth to [name of last child], who assisted in the delivery? Anyone else?” Options included doctor, nurse or midwife, auxiliary nurse or midwife, traditional birth attendant (TBA), relative or friend, other, or no one. We created a binary variable “use of a SBA”: coded as 1 (delivered by a SBA) if doctor, nurse or midwife, or auxiliary nurse or midwife was mentioned; and 0 (not delivered by a SBA) if otherwise.

#### Key independent variables: Socioeconomic status

We operationalize SES in this analysis as *education* and *wealth*. We examine education as a categorical variable (highest level of education attained by respondent). Wealth is measured in quintiles—calculated from a wealth index based on principal component analysis of variables on household assets [39].

#### Mediating variables: Proxies for the proximal determinants

*Perceived quality of care*: We operationalize perceived quality of care as an additive index based on women’s responses regarding whether they received nine ANC services at any point during their last pregnancy. The services included measuring weight and blood pressure, conducting urine and blood tests, prescribing iron supplements and an anthelminthic, vaccinating against tetanus, and instructing women on the signs of pregnancy complications and on where to go in case of a complication. Each question has a binary response (1 = Yes; and 0 = No). Although this index is arguably a better measure of actual quality than perceived quality, it is the best available proxy given the data collected.

The index (previous described elsewhere [26]) ranges from 0 to 9; the mean is 7.4. For this analysis, we dichotomized the index: good quality ANC (coded as 1) is receiving at least eight of the nine antenatal services (requiring a score above the mean); and poor quality ANC is receiving seven or fewer of the services (coded as 0). The assumption is that women who received good quality ANC (i.e. reported receiving more services) will have higher perceived quality of maternal health care than those who received poorer quality ANC, which will have a positive effect on their use of SBAs. However, considering this measure does not include questions on the interpersonal dimensions of quality [33,34] or disrespect and abuse [24,40], this measure may underestimate the role quality plays as a determinant of SBA utilization.

*Perceived accessibility*: We used place of residence—whether one lives in an urban or a rural area—as crude measure of perceived accessibility, absent better data regarding perceptions of access. Urban areas are defined as localities with 5000 or more persons, while rural areas are localities with less than 5000 persons [41]. Studies consistently find that urban women are more likely to use services than those in rural areas. Place of residence however reflects larger contextual factors beyond just physical access, including things like ability to pay, beliefs about use of health services, information availability, and quality of services [5,42]. Thus the urban effect will capture more than just access, including capturing some of the effects of the other potential mediators.

*Perceived Need*: The GMHS does not include data on perceived need for delivery services for all women; even though thinking it is “not necessary” is a common response when women who delivered at home are asked why they chose to deliver at home [29]. Since perceived need is influenced by current and past pregnancy and complication experiences, we constructed an index of perceived need based on six questions: whether women experienced a complication during the index pregnancy, had a multiple gestation (or a singleton), sought ANC for a check up or because of a problem (a proxy for early onset complications), had a past stillbirth, had a past miscarriage, and had a sibling who experienced a maternal death. In addition, we included two questions on whether the respondent has ever used contraception and knows where to get family planning as proxies for familiarity with the health system. The index ranges from 0 to 6 (no one responded positively to all 8 questions), with an average of 1.8. Because of the skewed distribution, we use it as a binary variable. Low need was a score of 0 or 1, coded as 0, and higher need was a score of 2 to 6, coded as 1. The assumption is that women with a higher need score will have a higher need for skilled birth attendance. But because perceived need is also influenced by health knowledge and sociocultural factors not captured in these data, this index also likely underestimates the effect of perceived need on behavior.

#### Control variables

We control for various factors that have been shown in the literature to be associated with use of SBAs: age, parity, marital status, and age at first union, which may tap into women’s autonomy; and religion and ethnicity, which may capture some sociocultural factors [5]. We also examine the frequency and timing of ANC visits and the type of ANC facility and provider.

### Statistical methods

Initial analyses involved descriptive statistics and examining the bivariate associations between the independent variables and the dependent variable using chi-squared tests. We then examined associations using multivariate binary logistic regressions to account for the effects of other factors. The models are built starting with the null model, then adding the key independent variables, the control variables, and finally the mediating variables. Mediating variables are those variables that explain the relationship between the key independent variables and the dependent variable. In this analysis the mediators are the proxies for the proximal determinants.

When the dependent variable is continuous, the mediated effect is the change in the coefficients of the key independent variables when the mediators are added to a linear regression model. However, because the addition of variables to a logistic model changes its scale, it is not accurate to consider the difference in the coefficients in nested logistic models as the magnitude of the mediated effect [43,44]. We therefore used the ‘khb’ rescaling method to calculate the magnitude of the mediated effects. In this method, the residual of the mediators are applied to the reduced model to fix the scale of the reduced model to that of the full model, so that the coefficients for the key independent variables can be compared across the nested models. The coefficients of the key independent variables in the models with the residuals of the mediators (the rescaled reduced model) are their total effects (c); and the coefficients of the key independent variables in the models with the actual mediators (the full model) are their direct effects (c’). The difference in the coefficients (c-c’) in the two models are the mediated effect, and the proportion of the total effect mediated is (c-c’)/c [43,45]. All the analyses are weighted using the sample weights provided with the data to account for the complex sampling design.

#### Robustness checks

We checked for collinearity and performed diagnostic tests to ensure the models were well specified. We used weighted single level logistic regression because we do not have the data to reconstruct the weights for multilevel analysis. To check the robustness of our results, we estimated unweighted multilevel (individual-level 1, cluster-level 2, and district-level 3) models with random intercepts for our final models using the “xtmelogit” command in Stata [46,47].

## Results

### Sample distribution

Table 1 shows the distributions of key study variables for the 97% of women who received ANC at least once during their last pregnancy (N = 4,868). These are similar to the distributions for the full sample, which have been described elsewhere [26,48]. The average woman in the sample is about thirty years old, has had about four pregnancies, and is married (72%). About a third of the women have no formal education and only about 8% have attended senior secondary school or higher. As expected, the analytic sample is almost evenly distributed between the five wealth quintiles. About two-thirds of the women live in rural areas. Of the women who attended ANC at least once, about 80% had four or more visits as recommended by WHO. About 61% received higher quality ANC (8–9 of the 9 services), and 58% have high need (2–6 of the need variables). A SBA assisted a little over half (57%) of the women at delivery—10% by doctors and 47% by nurses or midwives.

**Table 1:** Weighted distributions of key study variables for women who attended ANC at least once, GMHS, N = 4,868. Notes: ANC = Antenatal care; GMHS = Ghana Maternal Health Survey; JSS = Junior Secondary School; SSS = Senior Secondary School; SBA = Skilled Birth Attendant.

*Variables*	N	Proportion	[95% CI]	
**Highest education attained**				
None	1,588	0.330	0.296	0.364
Primary	1,072	0.221	0.202	0.239
Middle/JSS	1,804	0.375	0.345	0.404
Secondary/SSS/or higher	404	0.075	0.063	0.086
**Household wealth quintile**				
Poorest	1,024	0.207	0.177	0.236
Poorer	943	0.210	0.186	0.235
Middle	930	0.204	0.182	0.227
Richer	976	0.203	0.181	0.224
Richest	995	0.176	0.155	0.197
**Type of residence**				
Rural	2,967	0.648	0.617	0.679
Urban	1,901	0.352	0.321	0.383
**Perceived need score**				
Low (0–1)	2,029	0.423	0.398	0.448
High (2–6)	2,839	0.577	0.552	0.602
Mean	4,868	1.777	1.713	1.841
**ANC quality score**				
Low (0–7)	1,901	0.391	0.364	0.418
High (8–9)	2,967	0.609	0.582	0.636
Mean	4,868	7.406	7.322	7.490
**Delivery assisted by SBA**				
Yes	2,876	0.573	0.541	0.605
No	1,992	0.427	0.395	0.459

### Bivariate results

Table 2 shows the bivariate distributions for the key study variables by the delivery assistant. Use of SBAs increases with both education and wealth: 35% of those with no education used SBAs, compared to about 90% of those with secondary education or more; and 30% of the poorest women used SBAs, compared to 92% of the richest. Forty-one percent of women in rural areas were assisted by a SBA, compared to 87% of women in urban areas. About 64% and 65% of women with high need and those who received higher quality ANC respectively were assisted by a SBA, compared to 48% of those with low need and 45% of those who received lower quality ANC.

**Table 2:** Crosstabs of key variables by delivery assistant, GMHS, N = 4,868. Notes: See Table 1 notes for abbreviations.

*Variable*	Proportion assisted by a SBA	[95% CI]	
**Highest education attained**			
None	0.352	0.310	0.394
Primary	0.537	0.495	0.579
Middle/JSS	0.725	0.691	0.758
Secondary/SSS/higher	0.896	0.860	0.931
**Household wealth quintile**			
Poorest	0.298	0.252	0.344
Poorer	0.389	0.339	0.440
Middle	0.546	0.499	0.594
Richer	0.769	0.730	0.807
Richest	0.921	0.896	0.946
**Type of residence**			
Rural	0.412	0.374	0.449
Urban	0.870	0.842	0.898
**Perceived need score**			
Low (0–1)	0.479	0.435	0.524
High (2–6)	0.641	0.609	0.672
**ANC quality score**			
Low (0–7)	0.449	0.408	0.490
High (8–9)	0.652	0.620	0.685

### Multivariate logistic regression results

The results from the weighted multivariate logistic regression for use of SBAs are shown in Table 3. We present three sets of models, model 1 contains the key independent variables controlling for age, parity, marital status, age at first union, religion, and ethnicity; model 2 adds on the mediators; and model 3 adds on ANC frequency, timing, facility, and provider.

**Table 3:** Weighted binary logistic regression of use of SBAs on relevant predictors, GMHS, N = 4,868. Notes: See Table 1 notes for abbreviations; *p<0.05, ** p<0.01, *** p<0.001.

*Predictors*	*Odds of using a SBA*: *OR [95% CI]*								
	Excludes mediators^a^			Adds mediators^a^			Adds other ANC vars.		
*Key Independent variables*									
**Highest education**^b^									
None (ref)									
Primary	1.58***	[1.28	1.95]	1.46***	[1.18	1.80]	1.52***	[1.22	1.90]
Middle/JSS or higher	2.77***	[2.21	3.48]	2.41***	[1.91	3.03]	2.36***	[1.86	2.99]
** Household wealth**^b^									
Poorest (ref)									
Poorer/Middle	1.47**	[1.15	1.87]	1.19	[0.93	1.52]	1.1	[0.86	1.39]
Richer/Richest	6.45***	[4.74	8.76]	2.72***	[1.96	3.79]	2.17***	[1.56	3.03]
*Mediators*									
** Urban residence**				4.20***	[3.10	5.69]	3.96***	[2.88	5.45]
** Higher quality ANC**				1.47***	[1.25	1.73]	1.24*	[1.05	1.47]
** Higher need**				1.28**	[1.07	1.53]	1.26*	[1.04	1.51]
*Control variables***Current age in years**									
15-19yrs	0.50**	[0.32	0.79]	0.55*	[0.34	0.87]	0.65	[0.39	1.08]
20–24	0.63***	[0.48	0.82]	0.67**	[0.51	0.88]	0.68**	[0.51	0.90]
25–29 (ref)									
30–34	1.12	[0.85	1.48]	1.04	[0.79	1.37]	0.97	[0.73	1.28]
35–39	1.62***	[1.22	2.14]	1.41*	[1.07	1.87]	1.27	[0.95	1.69]
40-49yrs	1.26	[0.91	1.75]	1.15	[0.83	1.58]	1.06	[0.75	1.51]
** Parity**									
First child	1.82***	[1.36	2.43]	1.76***	[1.30	2.37]	1.71***	[1.26	2.32]
Second	1.11	[0.84	1.45]	1.07	[0.82	1.41]	1.07	[0.82	1.40]
Third (ref)									
Fourth	0.77	[0.58	1.03]	0.8	[0.60	1.07]	0.87	[0.65	1.16]
Five plus	0.71*	[0.53	0.95]	0.76	[0.57	1.02]	0.87	[0.65	1.18]
** Marital Status**									
Currently married (ref)									
Cohabitating	0.62***	[0.48	0.81]	0.63***	[0.48	0.83]	0.68**	[0.51	0.89]
Previously married	1.34*	[1.01	1.78]	1.19	[0.90	1.57]	1.25	[0.93	1.69]
Never married	1.09	[0.77	1.56]	0.98	[0.70	1.39]	1.04	[0.73	1.48]
** Married before 19years**	0.86*	[0.74	1.00]	0.9	[0.76	1.05]	0.9	[0.77	1.06]
** Religious affiliation**									
Orthodox Christian (ref)									
Other Christian	1.18	[0.95	1.47]	1.07	[0.87	1.33]	1.1	[0.88	1.38]
Moslem	1.13	[0.72	1.76]	0.98	[0.65	1.47]	0.97	[0.62	1.50]
Traditionalist /other	0.54***	[0.38	0.77]	0.50***	[0.35	0.72]	0.58**	[0.39	0.85]
** Ethnicity**									
Akan (ref)									
Ga/Dangme/Guan	0.70*	[0.50	0.98]	0.74	[0.53	1.02]	0.79	[0.57	1.11]
Ewe	0.58***	[0.42	0.80]	0.64**	[0.46	0.89]	0.67*	[0.48	0.92]
Mole-Dagbani/Hausa	0.59*	[0.38	0.91]	0.63*	[0.41	0.99]	0.63	[0.39	1.02]
Grussi/Gruma	0.68*	[0.47	0.99]	0.73	[0.49	1.08]	0.77	[0.52	1.14]
Other	1.08	[0.54	2.14]	1.03	[0.59	1.77]	1.03	[0.56	1.87]
*ANC variables*									
** Four or more ANC visits**							3.00***	[2.40	3.75]
** Trimester of first ANC**									
First trimester (ref)									
Second trimester							0.85	[0.72	1.01]
Third trimester							0.86	[0.54	1.38]
DK trimester							0.66	[0.16	2.68]
** ANC provider**									
Nurse/midwife (ref)									
Doctor							1.30*	[1.01	1.68]
All others							0.51*	[0.26	0.98]
** Type of facility**									
Gov't hosp./polyclinic (ref)									
Other Gov't facility							0.78*	[0.63	0.96]
Private /maternity home							1.03	[0.79	1.34]
Home/other/DK							0.46	[0.18	1.20]
Constant	0.46***	[0.31	0.69]	0.36***	[0.23	0.56]	0.20***	[0.12	0.34]
N	4,868			4,868			4,868		

^a^ The mediators are the proxies for the proximal determinants in our conceptual model (residence, ANC quality, and need).

^b^ The education and wealth categories regrouped into 3 groups for the multivariate analysis

All three models show that use of SBAs still increases with education and wealth net of other factors. Women with a middle school education or more and those in the highest wealth quintiles have over two times higher odds of using a SBA than those with no education and those in the lowest wealth quintiles respectively. The effects of the mediators are also significant net of other factors. Women who live in urban areas have about four times higher odds of using a SBA than those living in rural areas. Women who received higher quality ANC have about 47% higher odds of using a SBA than those who received lower quality ANC; and those with higher perceived need have about 28% higher odds of using a SBA than those with lower perceived need. We also find that women ages 20–24 are less likely to use a SBA than older women, while primiparous women are more likely to use a SBA than women with higher order births. Women who are cohabiting, those who belong to the Traditional/other religion group, and those of Ewe, Mole-Dagbani or Hausa ethnicity are less likely to use SBAs than those who are married, Christian, and Akan respectively.

The other factors that are positively associated with using a SBA in model 3 are attending ANC four or more times and receiving ANC from a doctor (compared to a nurse). Also, women who received ANC in lower-level facilities like health centers and health posts are less likely to use a SBA at delivery than those who did so in higher-level government facilities like hospitals or polyclinics. The mediation analysis is based on models 1 and 2. We exclude the ANC frequency, timing, facility, and provider from the mediation models because these variables are all associated with SES, rural/urban residence, and quality of ANC, as in previous studies [26,49,50]. In addition, they are associated with our perceived need measure—women with high need are more likely to start ANC early and be seen by doctors in higher-level facilities. Because these variables lie in the mediation pathway from SES to use of SBAs and are theoretically antecedent to ANC quality and perceived need, including them significantly reduces the magnitude the effects mediated by ANC quality and perceived need (although they are still significant).

### Mediation results

The results for the mediation analysis are shown in Table 4. Our measures of perceived access, perceived need, and perceived quality of care account for about 23% of the difference between women with no education and those with only primary school education and about 55% of the difference between women in the lowest wealth quintile and those in the middle wealth quintiles. In general, urban residence has the largest contribution to the mediated effect, which is not surprising because of its contextual nature, followed by quality, and then need—except for the difference between primary and no education where need has a slightly bigger effect.

**Table 4:** Mediation analysis using weighted binary logistic regression of use of SBAs and the ‘khb’ rescaling method.

	Mediated effect			% of total effect mediated by			
	*Coef*.	*Std_Err*	*P-value *	*Urban*	*ANC quality*	*Need*	*All 3 mediators*
**Highest Education**							
None (ref)							
Primary	0.11	0.04	0.01	13.79	3.85	5.10	22.74
Middle/JSS or higher	0.21	0.04	0.00	12.71	3.81	2.91	19.42
**Household wealth Index**							
Poorest (ref)							
Poorer/Middle	0.21	0.04	0.00	45.80	7.90	1.81	55.50
Richer/Richest	0.99	0.07	0.00	45.27	3.13	1.22	49.61

### Sensitivity results

For the most part, the findings from the unweighted multilevel logistic models are essentially the same in direction and significance of associations, and comparable in magnitude of the associations to the results from the weighted single level models presented. One exception is that the difference between those with no education and those with a primary education is not significant when the mediators are added to the multilevel model, where as there is still a significant difference in the single level model—potentially because standard errors tend to be smaller in single level models when data are clustered. The models using the full sample (including the 3% who did not attend any ANC) are essentially the same as that for the sample restricted to only women who had at least one ANC visit. In this model, quality of care is scored zero for those who did not attend ANC and an indicator variable is included in the model for whether or not the person received any ANC.

## Discussion

In this paper, we propose the DiSBA framework: a conceptual model for understanding sources of disparities in the use of SBAs, which posits perceived need, accessibility, and quality as the three proximal factors that affect use of SBAs; and apply it to the case of Ghana. We find that our measures of perceived need, access, and quality do account for some of the SES differences in the use of SBAs, although the effects mediated by quality and need are smaller than what we expect based on the results of several qualitative studies. This is likely because our measures do not adequately capture all dimensions of need and quality as previously discussed. In addition, the larger contribution of urban residence, which we use as a measure of access, likely, also captures some dimensions of need and quality.

Our findings for the effects of education, wealth, and urban residence are consistent with the findings from studies that examine the determinants of use of SBAs or facility deliveries [16,5,6,29,51]. Also, some of the variables we use to construct our perceived need index, like whether or not a woman has a complication and prior use of contraception, have been found to be determinants of use of SBAs in other studies [5,52–54]. In addition, although very few quantitative studies have examined quality of care as a determinant of MH service utilization, nearly all qualitative studies of MH service utilization mention quality of care as an important factor, with poor staff attitudes as a recurrent problem [18–20,24,35–37]. Our finding on the effect of quality differs from that of Stekelenburg et al. (2004) who found no effect of perceived quality of antenatal care on facility delivery in a rural district in Zambia. This is potentially because our measure of perceived quality of ANC captures more than just satisfaction, which is usually very high in surveys [30,55]. That women who reported receiving more services are more likely to use SBAs is not unexpected—ANC should be an opportunity to help women prepare for skilled attendance at delivery.

Our study goes beyond simply identifying determinants of utilization to understanding how distal factors affect use of SBAs through more proximal factors. To our knowledge, no other study has quantitatively examined the factors underlying SES disparities in the use of SBAs. While we do not place heavy emphasis on the magnitude of the effects in this analysis because of the weaknesses of our measures, our findings do suggest that the three proximal factors are important predictors of use of SBAs and do account for at least some of the SES disparities. Our findings also suggest that the important proximal factors may differ for different distal factors in different settings. For instance, we find that accessibility (urban residence) accounts for a larger proportion of the wealth differences in the use of SBAs in Ghana than the education difference, while need plays a bigger role in the education difference than the wealth difference.

Quality of care however has a relatively similar contribution for both the education and wealth differences—though more so for the difference between the poorest and middle wealth groups than the other differences. The contribution of ANC quality relative to accessibility and need is especially important because it supports our hypotheses that SES disparities in quality of ANC are also contributing to some of the SES disparities in the use of SBAs. This implies that bridging the SES disparities should go beyond educating women on the need to use SBAs and increasing accessibility of services to improving the quality of care provided to all women regardless of their SES. In addition the significant effect of ANC quality among women who attend some ANC suggest that improving quality of ANC will help reduce the coverage gaps between ANC attendance and skilled delivery care.

### Strengths and limitations

A major strength of this paper is that it uses a theory-based approach to data analysis. We propose a conceptual model to help understand the factors underlying SES disparities and illustrate how it can be used. The DiSBA framework can be adapted to examine the sources of other disparities including place of residence and age, as well as for other maternal health services. Such theory-based analysis will help address an important gap in the MCH literature, which is the dearth of quantitative studies exploring the mechanisms underlying various associations with use of maternal health services.

The main limitation is that we have had to use proxy measures for the proximal determinants, which may not adequately capture all dimensions. It would also have been preferable to have data on women’s existing comorbid medical conditions or her psychological state to determine if they had a role in her care seeking. However, such data were not available. GMHS data are cross-sectional, which limits causal inference; and recall and social desirability bias are potential problems, since the data are based on self-report. The age of the data is also a limitation. Nonetheless, this dataset has an advantage over the DHS of including a nationally representative sample of women who had a birth (live or stillbirth) in the five years preceding the survey, which reduces the chances of excluding women who received the worst care, as these women may be more likely to have a stillbirth.

## Conclusions

We have provided a theoretical framework to guide analysis to help improve our understanding of the sources of disparities in use of SBAs in low-resource settings. Next steps include developing and validating instruments to measure perceived need for maternal health services; perceived accessibility (physical, economic, and social), and perceived quality of care, capturing perceptions of disrespect and abuse. Such instruments should be incorporated into national surveys to allow for the collection of national data on proximal determinants.

Directly collecting data on women’s perception of need, access, and quality of care and the factors that influence these perceptions—especially actual accessibility (distance and cost of reaching and receiving services) and direct measures of quality of care—will be invaluable to understanding the factors underlying the persistent disparities in the use of maternal health services, particularly deliveries with skilled birth attendants. Such analysis will help elucidate the important underlying factors in different settings to ensure that programs to increase use of skilled attendants and other maternal health services target the most important underlying factors in each context. Until we have these better measures, our findings suggest that more efforts are needed not just to increase access and educate women on the need to use SBAs, but also to increase quality of care during any encounter women have the health system. Reducing disparities in access and quality of care, especially, are essential to reducing the SES disparities.
